# Regression patterns of uveal melanoma after iodine-125 plaque brachytherapy

**DOI:** 10.1186/s12886-021-01898-3

**Published:** 2021-03-16

**Authors:** Rui Fang, Heng Wang, Yang Li, Yue-Ming Liu, Wen-Bin Wei

**Affiliations:** 1 Beijing Tongren Hospital, Beijing, 100730 China; 2 Capital Medical University, Beijing, 100730 China; 3 Beijing Tongren Eye Center, Beijing, China; 4 Beijing key Laboratory of Intraocular Tumor Diagnosis and Treatment, Beijing, China; 5 Beijing Ophthalmology and Visual Sciences Key Lab, Beijing, China; 6 Medical Artificial Intelligence Research and Verification Laboratory of the Ministry of Industry and Information Technology, Beijing, China

**Keywords:** Uveal melanoma, Plaque radiotherapy, Tumor regression, Prognosis

## Abstract

**Background:**

Tumor regression of uveal melanomas (UMs) after radiotherapy has been reported as a valuable prognostic factor for metastasis and metastatic death. But its effect on prognosis is questionable. The purpose of this study was to summarize the regression features of uveal melanoma after iodine-125 plaque brachytherapy and the relationship with prognosis.

**Methods:**

Adult uveal melanoma patients who only received iodine-125 plaque brachytherapy between December 2009 and March 2018 at the Beijing Tongren Hospital, Capital Medical University were enrolled in this study. The regression rate was calculated as the percent change in tumor height, and each eye was classified for four main regression patterns: Decrease (D), Stable (S), Others (O), and Increase (I), according to the trend of height change. Statistical analysis was performed using one-way ANOVA and chi-square test, univariate and multivariate logistic regression, and Kaplan-Meier analysis.

**Results:**

A total of 139 patients was included in the study. The median follow-up was 35 months. Regression patterns status was pattern D in 65 tumors (46.8%), pattern S in 50 tumors (36.0%), pattern O in 6 tumors (4.3%), and pattern I in 18 tumors (12.9%). Reductions of tumor mean height for each follow-up visit were 5.26% (3 months), 10.66% (6 months), 9.37% (12 months), and 14.68% (18 months). A comparison (D vs. S vs. O vs. I) revealed the preoperative height of pattern I was significantly lower than the pattern D, S and O (mean: 7.24 vs. 7.30 vs. 6.77 vs. 5.09 mm, respectively; *P* = 0.037). LBD (largest basal diameter) was strongly associated with the metastasis (*P* = 0.03). However, an association between the tumor regression and subsequent melanoma-related metastasis and mortality could not be confirmed (*P* = 0.66 and *P* = 0.27, respectively). The tumor regression rate increased with increasing tumor height (*P* = 0.04) and decreased with increasing of LBD (*P* = 0.01).

**Conclusion:**

Our study showed a lack of association between the prognosis and the regression of uveal melanomas following I-125 plaque radiotherapy. The LBD and original height of the tumor have predictive value in tumor regression rate, and LBD was positively associated with metastasis.

**Supplementary Information:**

The online version contains supplementary material available at 10.1186/s12886-021-01898-3.

## Introduction

In 1984 Cruess et al. [1] firstly described the regression characteristics of uveal melanomas treated by Cobalt-60 plaque brachytherapy, showing the obvious heterogeneity of tumor regression patterns after radiotherapy. In 1987, Augsburger [2] and his colleagues found the rate and extent of tumor regression are unfavorable signs of the prognosis of the affected patients for subsequent development of the clinical metastatic disease. In 2004, Kaiserman et al. [3] got the consistent conclusion that the patients whose tumors regressed rapidly and completely were more likely to be dying of the metastatic disease.

These studies changed people’s previous standpoint that patients whose tumors regressed quickly and completely after irradiation tend to have a favorable systemic outcome than patients whose tumors regressed slowly and less completely [2]. Many of the related studies reported that the clinical tumor regression after radiation treatment to be an independent significant prognostic factor [4, 5]. However, not all authors support this view [1, 6–9], the prognostic value of tumor regression is still controversial.

Most of these researches focused on Euramerican countries. However, comparable research in Asian countries, including China, is scarce. Many prior studies have demonstrated that the white population of European and American countries have a higher incidence of uveal melanoma as compared to Asian countries [10]. Despite the racial differences, methods of treatment, and treatment guidelines of different countries are not entirely identical, which also contribute to the differential tumor responses to brachytherapy. To verify whether the tumor regression rate can be a prognostic factor, a better understanding of patient characteristics in our country is needed. Our study aimed to summarize the regression features of uveal melanoma after iodine-125 plaque brachytherapy in Chinese patients, and the relationship with prognosis.

## Patients and methods

This study was approved by the ethical committee of Beijing Tongren Hospital of Capital Medical University. Research adhered to the tenets of the Declaration of Helsinki. All subjects involved in the study are adults, more than 18 years old, and written informed consent was obtained from all subjects.

A retrospective review was performed on the medical records of posterior uveal melanoma patients who were treated with iodine-125 plaque brachytherapy by one physician (Dr. Yueming Lui) at the Beijing Tongren Hospital, Capital Medical University between December 2009 and March 2018. The standard dose of irradiation was 100 Gy to the apex of the tumor. In general, we treat tumors whose height blow 10 mm by brachytherapy. However, some patients who refused any other treatment and insisted on brachytherapy despite the physician’s reservations are also treated by brachytherapy. We excluded patients with iris or iridociliary melanomas and patients with metastatic disease at the time of diagnosis from the study. Receiving other treatments or combined therapy during the follow-up period was also an exclusion criterion.

Patient age, gender, and involved eye were available for each patient’s record from the baseline patient interview. The presence of subretinal fluid, tumor thickness and largest tumor diameter, tumor shape (dome shape, mushroom shape, flat shape, lobulated and others defined as irregular shape) and visual acuity, photographs, and ultrasound records were collected from the preoperative medical records. Clinical follow-up of patients was performed at least every 3 months during the first year after radiation, half a year or annually thereafter when tumor growth is stable. During each visit, Best-corrected visual acuity (BCVA) and the intraocular pressure (IOP) were recorded, and B-scan ultrasonography, fundus image were performed, and metastatic screening (liver function tests and liver ultrasound or computed tomography) were checked once every 6 months. For those who developed metastatic cancer during follow-up, the time of diagnosis of metastasis was recorded.

Tumor regression was evaluated as a percentage change of initial tumor height measuring with B-scan ultrasonography. Based on the tumor height changes over time according to Rashid et al. [11], each eye was classified for four main regression patterns: D (decrease; progressive decrease in height by at least 15% after brachytherapy), S (stable; less than 15% change in height), I (increase; progressive increase in height by at least 15%), and O (others, irregular change in height). Differences in baseline tumor height, LBD, presence of subretinal fluid, age at onset, eyes involved, gender, survival time and other clinical features between different regression patterns, and tumor regression rate were evaluated by one-way ANOVA, chi-square test and Kaplan-Meier analysis for the variance. Univariate and multivariate logistic regression was used to determine which factors had a statistically significant effect on tumor regression and prognosis. All calculations were performed in Statistical Package for the Social Sciences (SPSS) version 23 by International Business Machines Corporation, Armonk, New York, United States.

## Results

Demographics by tumor regression patterns (D vs. S vs. O vs. I) were shown in Table 1. One hundred thirty-nine patients were included in our study, 55.4% men, and 44.6% women. The median age at diagnostic was 46 years (range 37–53), the youngest patient was 19 years old and the oldest 72 years old. Both eyes were equally involved (50.3 and 49.7% for right and left eyes, respectively). Median follow-up time was 35 months (mean 39.58 mos, range 95 mos). The height of the tumors treated in our study was between 2.2 mm and a maximum of 12.6 mm (mean: 6.97). The mean basal diameter was 12.09 mm. Residual subretinal fluid was present in 105 eyes (75.5%). Tumour AJCC stages T1, T2, T3 and T4 occurred in 9 (6.5%), 73 (52.5%), 53 (38.1%) and 4 (2.9%), respectively. Since most of the tumors are in the same group according to COMS criteria, we divide the size of the tumor into larger tumors (L: height more than 10 mm), medium-sized tumors (M: height between 5.1 and 9.9 mm), and small-sized tumors (S: height less than 5 mm), containing 17(12.2%), 83(59.7%) and 39(28.1%), respectively. As for tumor shape, 69 (49.6%) were the mushroom shape, 2 (1.4%) flat shape, 60 (43.2%) dome shape, and 8 (5.8%) irregular shape. Trends of the tumor regression are classified into pattern D (*n* = 65 [46.8%]), S (*n* = 50 [36.0%]), O (*n* = 6 [4.3%]), and I (*n* = 18 [12.9%]).

**Table 1:** Regression patterns: D, S, O, I: patient demographics

Demographics	Regression patterns, No. (%)				*P* Value	Total (*n* = 139)
	D (*n* = 65)	S (*n* = 50)	O (*n* = 6)	I (*n* = 18)		No,%
Age (yrs)						
mean ± SD	44.69 ± 10.82	45.85 ± 13.10	51.17 ± 7.68	46.72 ± 11.79	*P*>0.05	45.65 ± 11.68
median	44	46	53	47		46
95%CI	[42.01 47.37]	[42.12 49.56]	[43.11 59.23]	[40.86 52.59]		
Gender						
male	34 (52.3)	29 (58.0)	4 (66.7)	10 (55.6)	*P*>0.05	77 (55.4)
female	31 (47.7)	21 (42.0)	2 (33.3)	8 (44.4)		62 (44.6)
Involved eye						
right	29 (44.6)	28 (56.0)	2 (33.3)	11 (61.1)	*P*>0.05	70 (50.3)
left	36 (55.4)	22 (44.0)	4 (66.7)	7 (38.9)		69 (49.7)
Follow-up (mos)						
mean	44	33	38	44	*P* = 0.005	39.58
median	36	32	33	40		35
Outcome						
Metastasis	7 (10.8)	6 (12.0)	2 (33.3)	1 (5.6)	*P*>0.05	16 (11.5)
Death (resulting from metastasis)	6 (9.2)	4 (8.0)	2 (33.3)	0	*P*>0.05	12 (8.6)
Time to metastasis (mos)						
mean	38.29	21.5	30	43	*P*>0.05	31.25
median	33	24.5				28.5
Time to death (mos)						
mean	51	31	33		*P*>0.05	42
median	52	32				35
Time from metastasis to death (mos)						
mean	14	13	4		*P*>0.05	11.58
median	10	11				6.5

**Fig. 1:**
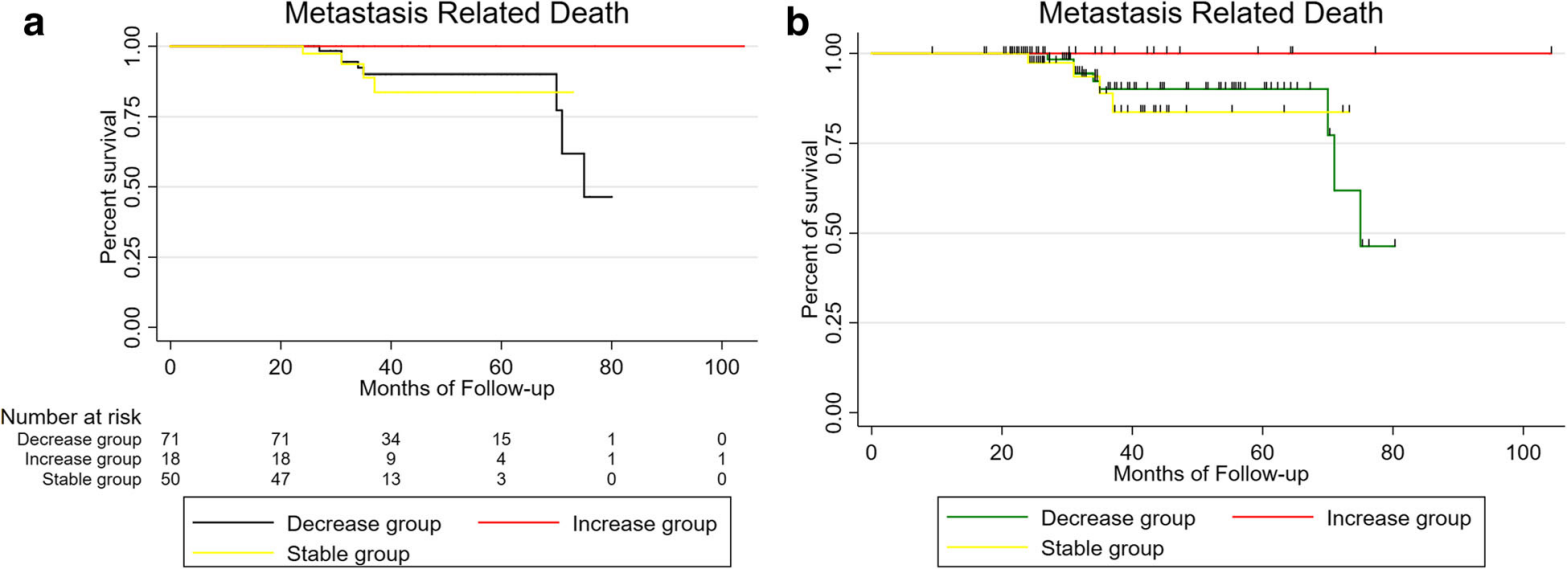
Kaplan-Meier metastasis free UM patients survival curve after iodine 125 brachytherapy by tumor regression patterns (D vs. S vs. I), expressed in months of follow up

**Fig. 2:**
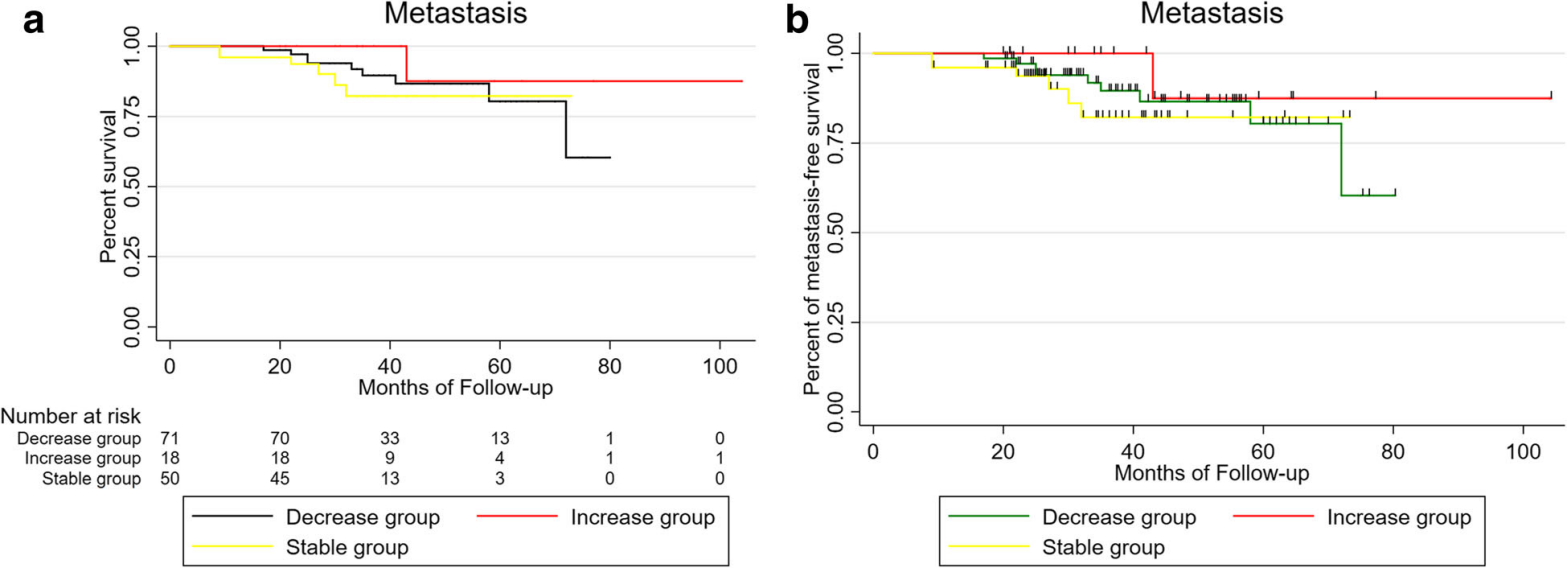
Kaplan-Meier analysis of survival curve for patients with uveal melanoma after iodine 125 plaque radiotherapy by tumor regression patterns(D vs. S vs. I), expressed in months of follow up

There was no difference in onset age (mean: 44.69 vs. 45.85 vs. 51.17 vs. 46.72 years, respectively; *P* = 0.59), gender (male: 52.3% vs. 58% vs. 66.7% vs. 55.6%, respectively; *P* = 0.87), or affected eye (right eye: 44.6% vs. 56% vs. 33.3% vs. 61.1%, respectively; *P* = 0.39) among different regression patterns. Metastasis and death rate of tumor regression patterns D/ S /O/I were 10.8% /12.0% /33.3% /5.6 and 9.2% /8.0% /33.3% /0, respectively. Results among different regression patterns(D vs. S vs. I), there were no statistical significance of metastasis (*P* = 0.5) and death (*P* = 0.27) (Figs. 1, 2), O group was excluded because of its small sample size.

**Fig. 3:**
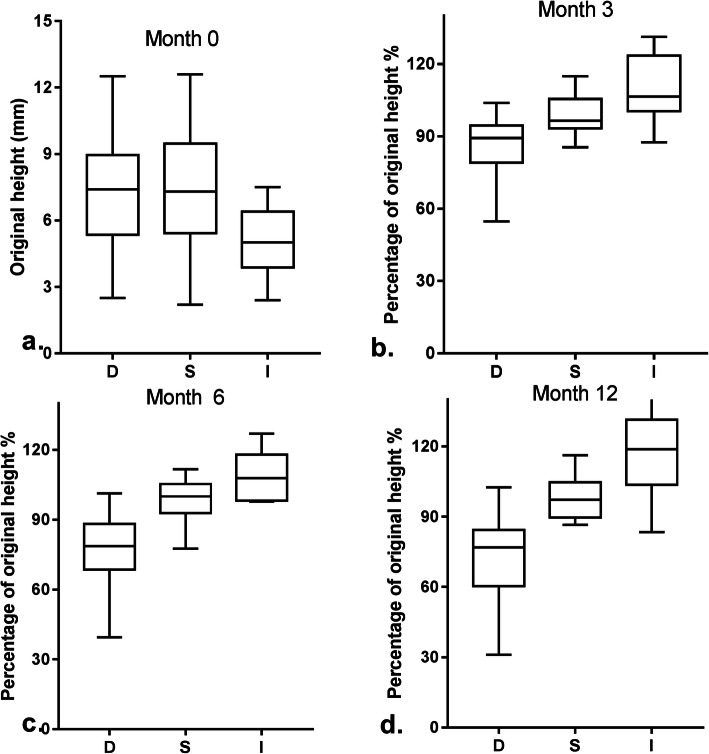
Pre-brachytherapy tumour height of uveal melanoma (**a**), percent of initial tumor thickness changes after iodine 125 brachytherapy in month 3 (**b**), month 6 (**c**), month 12 (**d**) by tumor regression patterns (D vs. S vs. I)

Clinical characteristics of UM by tumor regression patterns are described in Table 2. A Comparison (D vs. S vs. O vs. I) revealed the preoperative height of pattern I was significantly lower than the pattern D, S and O (mean: 7.24 vs. 7.30 vs. 6.77 vs. 5.09 mm, respectively; *P* = 0.037) (Fig. 3a). Differences of tumor shape between regression patterns indicates statistical significance (mushroom shape: 49.2% vs.44.0% vs. 50% vs. 16.6%, respectively; *P* = 0.025). However, less significant results found in diameter between different regression patterns (mean: 11.48 vs. 12.44 vs. 12.00 vs. 13.31 mm, respectively; *P* = 0.09). The same was found for residual subretinal fluid (persistent: 75.4% vs. 78.0% vs. 83.3% vs. 66.7%, respectively; *P* = 0.77), AJCC staging (T2: 55.4% vs. 48.0% vs. 50.0% vs. 55.6%, respectively; *P* = 0.94), and tumor size (M: 63.1% vs. 58.0% vs. 66.7% vs. 50.0%, respectively; *P* = 0.20).

**Table 2:** Regression patterns: D, S, O, I: tumor characteristics

Tumor characteristics	Regression patterns, No. (%)				*P* Value	Total (*n* = 139)
	D (*n* = 65)	S (*n* = 50)	O (*n* = 6)	I (*n* = 18)		No. (%)
Tumor height, mm						
mean ± SD	7.24 ± 2.37	7.30 ± 2.58	6.77 ± 1.83	5.09 ± 1.47	*P* = 0.005	6.97 ± 2.43
median	7.4	7.3	6.85	5		6.7
95%CI	[6.65 7.83]	[6.57 8.04]	[4.85 8.68]	[4.36 5.82]		
Tumor diameter, mm						
mean ± SD	11.48 ± 2.72	12.44 ± 2.94	12.00 ± 3.74	13.31 ± 3.45	*P*>0.05	12.09 ± 2.98
median	11.5	12.3	12.1	12.5		11.9
95%CI	[10.81 12.16]	[11.60 13.27]	[8.08 15.92]	[11.59 15.03]		
Subretinal fluid						
absent	16 (24.6)	11 (22.0)	1 (16.7)	6 (33.3)	*P*>0.05	34 (24.5)
persistent	49 (75.4)	39 (78.0)	5 (83.3)	12 (66.7)		105 (75.5)
AJCC staging						
T1	4 (6.2)	3 (6.0)	0	2 (11.1)	*P*>0.05	9 (6.5)
T2	36 (55.4)	24 (48.0)	3 (50)	10 (55.6)		73 (52.5)
T3	24 (36.9)	21 (42.0)	3 (50)	5 (27.8)		53 (38.1)
T4	1 (1.5)	2 (4.0)	0	1 (5.5)		4 (2.9)
Tumor Size						
S	16 (24.6)	12 (24.0)	2 (33.3)	9 (50.0)	*P*>0.05	39 (28.1)
M	41 (63.1)	29 (58.0)	4 (66.7)	9 (50.0)		83 (59.7)
L	8 (12.3)	9 (18.0)	0	0		17 (12.2)
Tumor Shape						
mushroom shape	32 (49.2)	22 (44.0)	3 (50.0)	3 (16.6)	*P* = 0.02	60 (43.2)
flat shape	1 (1.5)	0	0	1 (5.6)		2 (1.4)
dome shape	30 (46.2)	27 (54.0)	2 (33.3)	10 (55.6)		69 (49.6)
irregular shape	2 (3.1)	1 (2.0)	1 (16.7)	4 (22.2)		8 (5.8)

Associated factors of tumor regression and prognosis are listed in Table 3. Only the terms of *P*<0.3 in univariate logistic regression can be encountered in the Table 3, and then were selected to analyze with multivariate logistic regression. Univariate logistic regression revealed no association between tumor regression rate and metastasis (*P* = 0.66) and death (*P* = 0.27). However, LBD was strongly associated with the metastasis (OR, 1.21 for each 1-mm change in diameter, 95%CI[1.02, 1.44], *P* = 0.03). Multivariate analysis showed tumor height (OR, 1.17 for each 1-mm increase, 95%CI[1.00, 1.37], *P* = 0.04), and LBD (OR, 0.83 for each 1-mm increase, 95%CI[0.73, 0.95], *P* = 0.01) independently associated with tumor regression.

**Table 3:** Prediction of tumor regression and prognosis

	Univariable logistic regression			Multivariable logistic regression		
	OR	95% CI	*P* Value	OR	95% CI	*P* Value
Prediction of tumor Regression						
Involved eye	1.74	[0.89, 3.40]	0.11	1.79	[0.89, 3.58]	0.1
height	1.09	[0.95, 1.25]	0.24	1.17	[1.00, 1.37]	0.04
LBD	0.87	[0.78, 0.98]	0.03	0.83	[0.73, 0.95]	0.01
Prediction of Metastasis						
LBD	1.21	[1.02, 1.44]	0.03	1.17	[0.98, 1.40]	0.09
SF	5.5	[0.70, 43.29]	0.105	3.62	[0.44, 30.01]	0.23
Age	0.97	[0.93, 1.02]	0.24	0.98	[0.93, 1.02]	0.36
Prediction of Death						
LBD	1.13	[0.93, 1.36]	0.22	1.12	[0.91, 1.39]	0.78
SF	3.86	[0.48, 31.07]	0.2	2.95	[0.34,25.35]	0.32
D regression	2.03	[0.58,7.09]	0.27	2.35	[0.64, 8.61]	0.2

The tumor regression outcomes at 3-, 6-, 12- and 18-months follow-up are summarized in Table 4 (52 patients had follow-up till 18 months). Reductions of tumor mean height for each follow-up visit were 5.26%(3 months), 10.66%(6 months), 9.37%(12 months) and 14.68%(18 months). Age and regression patterns were significantly associated with tumor regression rate. Patients who were younger than 46 regressed faster than those who were 46 or older (12 months: 15.33% vs. 3.53%, respectively; *p* < 0.05). Different from the reduction in the two patterns, tumors in pattern I grow to larger sizes at all the time points (+ 9.23% vs. + 8.22% vs. + 21.92% vs. + 22.48%, respectively), in line with our classification (Fig. 3b, c, d). The following features indicate no statistical significance with tumor regression: sex of patient(12 months in male vs female: 7.54% vs. 11.16%, *p* = 0.52), involved eye (12 months in left vs right: 10.06% vs. 8.75%, *p* = 0.81), subretinal fluid (12 months in absent vs. persistent: 7.99% vs. 9.96%, *p* = 0.75), tumor size (12 months in S vs. M vs. L: 3.34% vs. 11.49% vs. 15.05%, *p* = 0.33) and tumor shape (12 months in mushroom vs. dome vs. irregular: 11.68% vs. 9.37% vs. 0.45%, *p* = 0.55).

**Table 4:** Tumor regression: patient demographics and tumor features

Demographics and tumor features	Fellow-up time (Mean,%)				*P* Value
	3 month	6 month	12 month	18 month	
Age (yrs)					
≥46	5.49	10.09	15.33	20.1	*P*<0.05
	5.03	11.27	3.53	8.82	
Gender					
male	4.4	8.11	7.54	13.63	*P*>0.05
female	6.28	13.67	11.16	15.81	
Involved eye					
left	5.68	10.61	10.06	16.16	*P*>0.05
right	4.86	10.71	8.75	13.31	
Regression patterns					
D	13.62	22.78	27.31	33.24	*P*<0.05
S	1.17	1.1	2.47	1.41	
I	^a^9.23	^a^8.22	^a^21.92	^a^22.48	
Subretinal fluid					
absent	5.75	10.61	7.99	16.14	*P*>0.05
persistent	5.07	10.67	9.96	12.24	
Tumor Height					
≤ 5 mm	2.01	13.17	3.34	13.7	*P*>0.05
5-10 mm	5.82	9.72	11.49	12.79	
≥ 10 mm	9.76	10.19	15.05	21.88	
Tumor shape					
mushroom shape	7	11.74	11.68	17.05	*P*>0.05
dome shape	3.81	10.85	9.37	14.97	
irregular shape	2.21	1.78	0.45	^a^8.13	
mean regression extent	5.26	10.66	9.37	14.68	

^a^denotes increase percentage in original height

## Discussion

Despite effective local ocular tumor control for primary UM in the era of plaque radiotherapy, death resulting from the metastatic disease remains prevalent [12], with metastasis in nearly 50% of patients with large uveal melanomas, particularly to the liver. Once metastasis is clinically detected, the survival time of patients will be remarkably shortened. Identifying patients at high risk of UM-related metastasis is significantly important to improve the prognosis of patients with UM. Some studies indicate monosomy 3 and gene expression profiling are reliable prognostic indicator for metastatic potential in uveal melanoma [13, 14]. Compared with complex chromosome analysis, tumor regression of uveal meloma after plaque radiotherapy showed better clinical feasibility to evaluate the risk of metastasis. However, the prognostic value of tumor regression is still questionable.

**Fig. 4:**
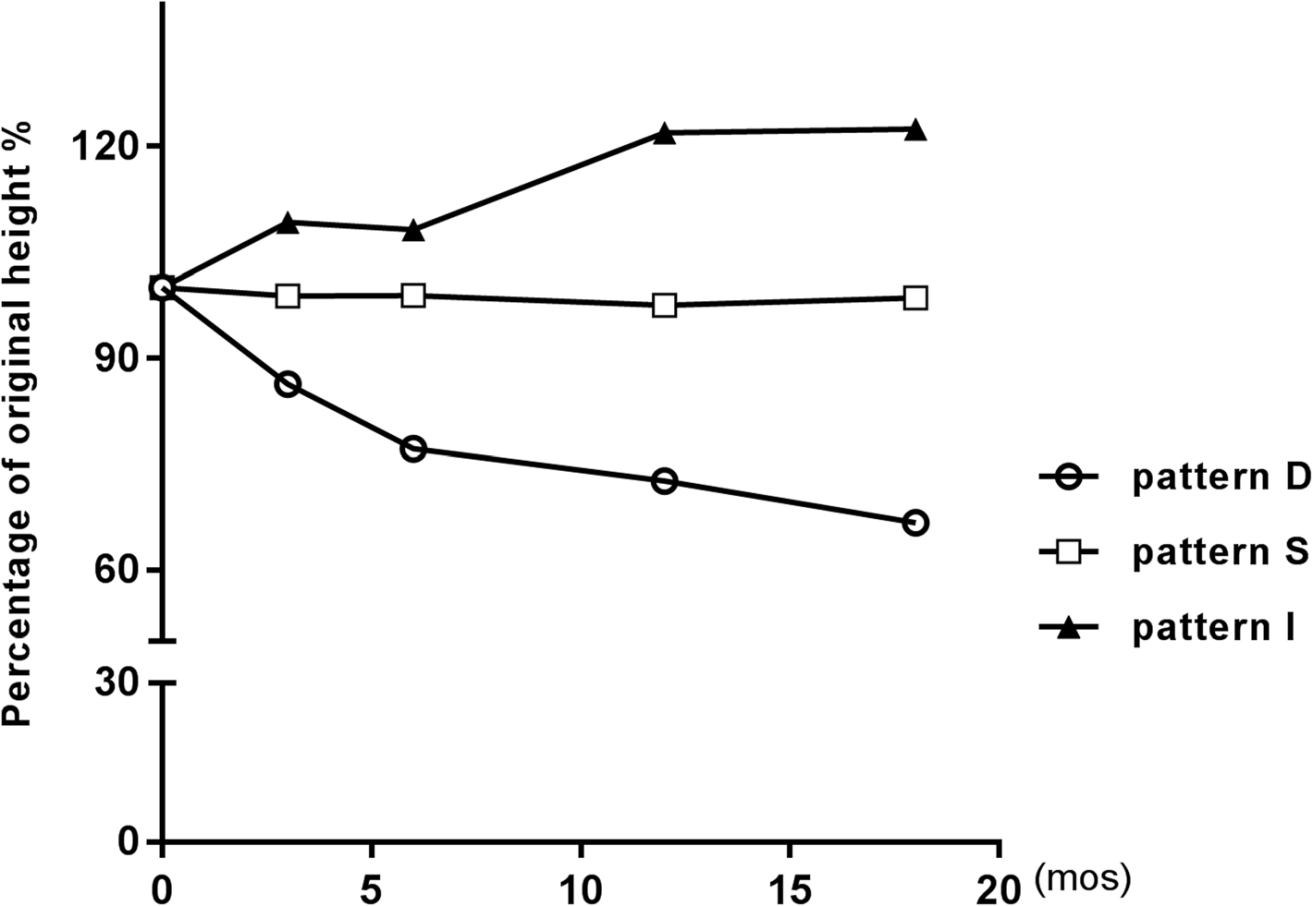
Graph of mean percent of initial tumor thickness versus duration of follow-up after iodine 125 brachytherapy for patients with uveal melanoma by tumor regression patterns (D vs. S vs. I)

The median age of uveal melanoma diagnosis is 59 to 62 years in the United States and Europe [15]. Our study indicates lower age at diagnosis, with a median age of 46 years. The incidence of uveal melanoma is higher in males compared with females, consistent with the previous studies [16]. Abramson et al. [5] analyzed 82 UM patients for tumor regression patterns, finding that no two uveal melanomas regressed exactly in the same pattern after brachytherapy. In their study, 70% regressed progressively (pattern D), 16% remained stable (pattern S), 12% increased in size (pattern I), and 2% showed other patterns (pattern O). 12.9% of uveal melanoma increased over time and 4.3% regressed in other patterns in our study, a very similar result. Conversely, we found that 36% of uveal melanomas remained stable and only 46.8% regressed in pattern D which is similar to Rashid’s study [11]. Cruess et al. [1] found mean reductions in height of 6 months, 12 months and 54 months after brachytherapy were 20, 30 and 50% compared with baseline. In our series, the height reduction of 3 months, 6 months, 12 months and 18 months were 5.3, 10.7, 9.4 and 14.7%, a discrepant result. A possible explanation for the different results is the use of different isotopes since the isotope used has been reported as an independent predictor of tumor regression [17]. And our clinicians also found that most tumors of uveal melanoma patients did not regress significantly after iodine-125 plaque brachytherapy compared with Western patients. The regression rates in each pattern were obviously different, pattern O was excluded in the comparison for its height change waxes and wanes. However, similar trends were observed for the three patterns. Tumor height decline/increase rapidly in the first 3 months after radiotherapy, followed by slower change later (regression rate in pattern D: 4.5 vs.3.8 vs. 2.3 vs. 1.8; %/month) (Fig. 4).

The tumor original heights were positively associated with tumor regression rate in agreement with Rashid’s [17] findings which showed larger tumors shrink significantly faster than smaller ones. Our study also found LBD remained the predominant and robust predictor of tumor regression rate, which was negatively associated with tumor regression rate. Also besides, LBD was strongly associated with the metastasis. The larger the LBD of the tumor, the more likely it is to metastasize. Onset age also has a place in the tumor regression rate. Abramson et al. [5] found age was an important predictor of survival of UM patients, instead of 46 years limit their used 60 years limit. In our study, patients who were younger than 46 regressed faster, albeit not verified in logistic regression analysis.

Augsburger [2] and Kaiserman [3] put forward a point that rapid regression of tumors after plaque brachytherapy is an unfavorable sign for UM patients. Inconsistent with their research, an association between the tumor regression and subsequent melanoma-related metastasis and mortality could not be confirmed in our study. We found, in agreement with Cruess’s [1] research, that the rate and extent of regression of the tumors in patients who subsequently developed metastatic melanoma and patients who remained well systemically were not appreciably different. There is no definite correlation between the rate and extent of post-irradiation shrinkage of one of these tumors and the patient’s prognosis for survival. Recently some researchers attempted to figure out the association between tumor regression rate and the status of 3 chromosome [6, 7, 18, 19] or prognostic gene expression profile (GEP) [8, 9], viewing from another Angle, to verify the prognostic value of tumor regression. Nevertheless, there are no conclusions regarding tumor regression rate and molecular characteristics. Some studies retorted that uveal melanomas with chromosome 3 monosomy showed faster and greater tumor regression after plaque radiotherapy and thermotherapy than melanomas with disomy 3, the authors believed tumor regression is an adverse prognostic factor as chromosome 3 monosomy, which is highly lethal [18, 19]. However, other investigators questioned the view, they had failed to turn up any evidence to confirm a relation between tumor regression and chromosome 3 monosomy [6, 7]. What is interesting is that some academics demonstrated that GEP class1 UM tumors tend to regress more rapidly than class2 tumors after plaque radiotherapy [8, 9]. Gene expression profiling (GEP) classifies patients according to metastatic risk: class 1 tumors have a low risk and class 2 tumors have a high risk of metastasis [20]. Seen from this angle, the faster rate of regression after radiation therapy may be a positive prognostic factor for survival associated with metastatic disease and death, contradicting with previous researches. In summary, studies relating to tumor regression, genetic features, metastasis and survival remain conflicting and do not arrive at a consensus. The current data can not distinguish whether the tumor regression rate after brachytherapy predicts metastasis, further study is required.

In conclusion, we have described the post brachytherapy regression features of uveal melanoma indicating initial tumor height and LBD are both powerful and valuable predictor for tumor regression, LBD is also positive with metastasis. Nevertheless, the association between tumor regression of UM after radiation and prognosis as yet undiscovered.

## Supplementary Information

**Additional file 1.**

## Data Availability

The datasets generated during the current study are available in the [raw data] excel file.
